# Platelet microparticle-mediated transfer of miR-939 to epithelial ovarian cancer cells promotes epithelial to mesenchymal transition

**DOI:** 10.18632/oncotarget.22136

**Published:** 2017-10-27

**Authors:** Meiling Tang, Lu Jiang, Yingying Lin, Xiaoli Wu, Kai Wang, Qizhi He, Xipeng Wang, Weiping Li

**Affiliations:** ^1^ Department of Gynecology, Shanghai First Maternity and Infant Hospital, Tongji University School of Medicine, Shanghai, China; ^2^ Department of Neurosurgery, Renji Hospital, School of Medicine, Shanghai Jiao-Tong University, Shanghai, China; ^3^ Central Laboratory, Shanghai First Maternity and Infant Hospital, Tongji University School of Medicine, Shanghai, China; ^4^ Department of Pathology, Shanghai First Maternity and Infant Hospital, Tongji University School of Medicine, Shanghai, China; ^5^ Department of Gynecology and Obstetrics, Xinhua Hospital, Affiliated with Shanghai Jiao Tong University, School of Medicine, Shanghai, China; ^6^ Department of Obstetrics and Gynecology, Renji Hospital Affiliated to Shanghai Jiao Tong University School of Medcine, Shanghai, China

**Keywords:** EOC, PMPs, epithelial-mesenchymal transition (EMT), miR-939, secretary phospholipase A2 type IIA (sPLA_2_-IIa)

## Abstract

Epithelial ovarian cancer (EOC) patients frequently suffer from thrombocytosis, which leads to a poor prognosis. However, the mechanism underlying platelet regulation of biological behavior in EOC remains unclear. The associations between clinicopathological characteristics and thrombocytosis in 171 EOC patients were studied, preoperative thrombocytosis was significantly associated with the stage, metastasis scope, level of preoperative CA125 and overall survival. When SKOV3 cells were cocultured with platelet microparticles (PMPs), the expression of molecules associated with epithelial-mesenchymal transition (EMT) was increased. The proliferation and migration of SKOV3 cells were also enhanced. Based on the miRNA microarray of the PMPs derived between thrombin-stimulating and apoptotic platelets, we demonstrated that over-expression or complete knockdown of miR-939 in the SKOV3 cells strengthened or weakened EMT. Secretory phospholipase A2 type IIA (sPLA_2_-IIa) has been shown to mediate PMPs intake by SKOV3 cells. The knockdown of sPLA_2_-IIa in SKOV3 cells verified that PMPs were involved in crosstalk during the regulation of cancer cells by transferring miRNA. This study revealed an important role for PMPs in the crosstalk of platelets and cancer cells through miR-939 shedding mediated by sPLA_2_-IIa, which enables EOC to undergo EMT and enhances cancer progression. Our findings pave the way for developing a novel therapeutic strategy for EOC targets such as PMPs, miR-939 or sPLA_2_-IIa.

## INTRODUCTION

Epithelial ovarian cancer (EOC) accounts for 90% of all ovarian cancers and has become the fifth leading cause of cancer-related death among women [1]. Due to the lack of efficient screening programs, approximately 70% of patients are diagnosed at an advanced stage (FIGO stage III and IV), resulting in a 30% of 5-year overall survival (OS) rate [2]. Some of the various clinicopathologic characteristics observed in advanced EOC include wide spread peritoneal metastases and thrombocytosis. However, the relationship between abundant platelets and tumor metastasis remains unclear.

The association between coagulation and cancer dates back to 1865. Some EOC patients present with thrombocytosis as their initial symptom [3]. Clinical data indicate that paraneoplastic thrombocytosis is a prevalent phenomenon in patients with many types of solid tumors (gastrointestinal, lung, breast, or ovarian cancer) associated with cancer cell progression, such as angiogenesis, invasion, metastasis, poor survival, chemoresistance, and recurrence [4, 5]. EOC treatments targeting platelets (anti-IL6 or thrombopoietin receptor therapy) could significantly inhibit the progression of EOC [2].

One of the major mechanisms involved in platelet-cancer cell interactions is tumor cell-induced platelet-aggregation (TCIPA) [6]. TCIPA leads to the formation of platelet cancer aggregates that adhere to the endothelium and may cause distant embolization of the microvasculature, which stimulates angiogenesis [7, 8]. The secretory function of platelets also plays a very important role in stabilizing tumor vasculature and preventing intratumoral hemorrhages. Platelet-derived factors, including α granules and dense granules, could promote cancer cell proliferation and metastasis and maintain angiogenic microvessel integrity [9].

Most recently, interest in from 0.1 to 1.0μm PMPs secreted from activated platelets is increasing. Because PMPs contain platelet-derived bioactive molecules including miRNA, growth factors, and other signaling molecules. PMPs uptake induces functional changes in target cells. For example, PMP miRNAs can silence mRNA targets. Therefore, we compared the mRNA profiles of thrombin-stimulating platelet-derived PMPs against apoptotic platelet PMPs using a microarray assay to screen functional miRNA associated with EOC malignant behavior. In addition, secretory phospholipase A2 type IIA (sPLA_2_-IIa) is secreted by platelets and can induce PMPs internalized in neutrophils [10, 11]. Thus, we hypothesized that sPLA_2_-IIa is likely a key factor participating in the process of PMPs uptake by cancer cells.

In this study, we demonstrated that the selective uptake of PMPs by EOC could promote the initiation of EMT and ultimately support metastasis in ovarian cancer. To clarify the role of PMPs in EOC progression, we investigated the mechanism of PMPs containing miRNA engaging in crosstalk between platelets and EOC cells.

## RESULTS

### EOC patients exhibited a high level of platelets

**Figure 1:**
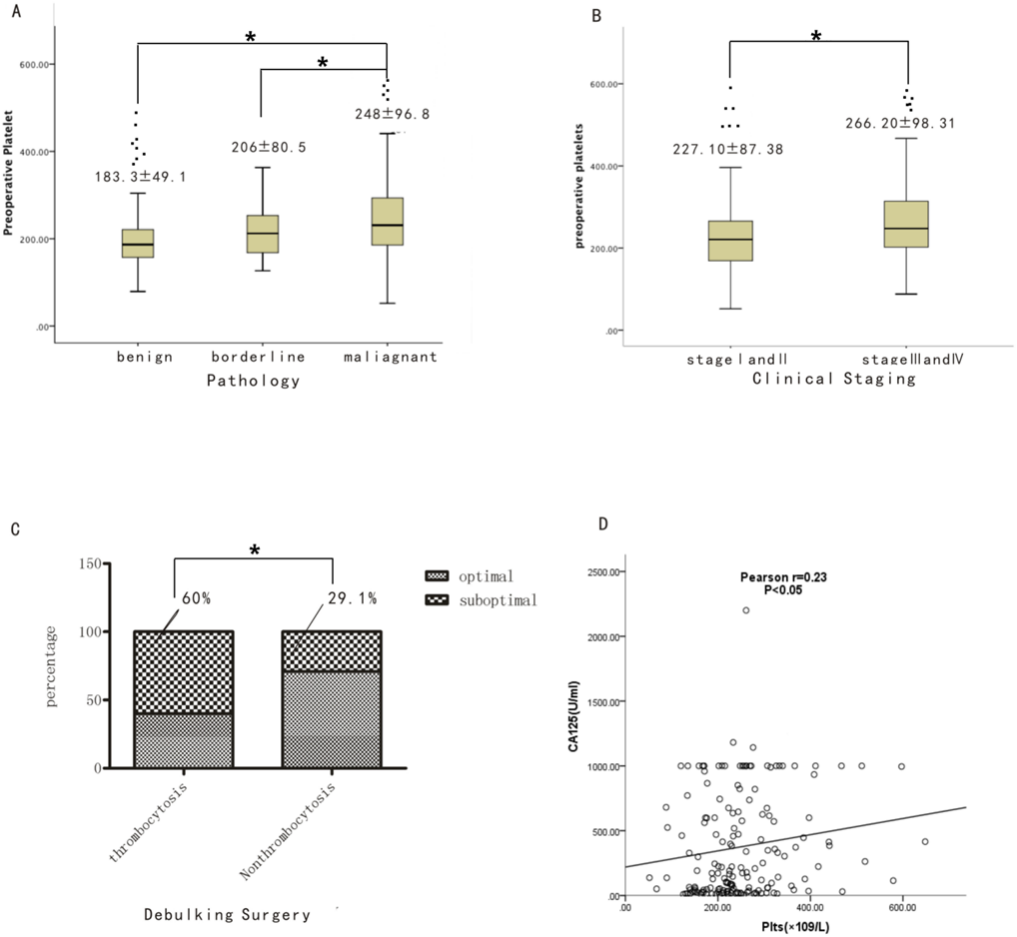
Comparison of EOC patients with thrombocytosis and non-thrombocytosis **(A)** Comparison of preoperative platelet count among benign epithelial ovarian tumor, borderline ovarian tumor and EOC (All P <0.05). **(B)** Comparison of preoperative platelets count between EOC at early stage (I and II) and advanced stage (III and IV) (P<0.05). **(C)** Percentage of optimal debunking surgery between EOC with thrombocytosis and EOC without it (P<0.05). **(D)** Linear regression analysis of CA125 levels with preoperative platelets in EOC (P<0.05).

The clinical demographic data are listed in Supplementary Table 1. Of the 3 groups with ovarian tumors, the platelets in the EOC patients were elevated significantly (183.3 × 10^9^/L in benign, 206.9 × 10^9^/L in borderline, 248.0 × 10^9^/L in EOC, p < 0.001, Supplementary Table 1, Figure 1A) according to blood cell analyses. With threshold platelet counts of 350 × 10^9^/L and 400 × 10^9^/L, the rate of thrombocytosis was 11.7% (20/171) and 7% (12/171) in the EOC patients in the Chinese population (Supplementary Table 1). If the general criterion of thrombocytosis of 400 or 450 × 10^9^ /L was selected, the rate of thrombocytosis would be relatively low. Thus, 350 × 10^9^ /L was selected for this study. Stage III and stage IV EOC patients exhibited significantly higher levels of preoperative platelets than stage I and stage II EOC patients (Figure 1B).

### High levels of platelets predicted an advanced stage, optimal surgery and overall survival

As shown in Supplementary Table 2, no significant differences in age, hemoglobin, lymphocyte count, prothrombin time(PT), tumor markers after the third cycle of chemotherapy, or the preoperative platelet to postoperative platelet ratio (defined as the preoperative platelet count divided by the platelet count after three cycles of primary adjuvant chemotherapy) were noted among the 171 EOC cases with thrombocytosis and non-thrombocytosis. With respect to the clinical demographic data, signs of ascites (P=0.029) were remarkable in the EOC patients with thrombocytosis, which suggests that more advanced clinical symptoms were associated with thrombocytosis. In surgical pathology, 60% of the EOC patients with thrombocytosis exhibited a residual tumor size greater than 1 cm after the debulking surgery compared with 29.1% of the EOC patients with non-thrombocytosis. This difference was statistically significant (p = 0.01) (Figure 1C). Thus, more cases of EOC with thrombocytosis were observed in the advanced stage (FIGO III and IV) compared with the of EOC patients without thrombocytosis (P=0.029) (Figure 1C). With respect to organ involvement during the operation, more organs, including the omentum (p = 0.006), diaphragmatic surface (p = 0.018) and peritoneum (p = 0.016), exhibited a higher incidence of involvement in the EOC patients with thrombocytosis compared with patients without thrombocytosis. Besides, the level of preoperative CA125 was positively correlated with preoperative platelet (p < 0.05) (Figure 1D).

**Figure 2:**
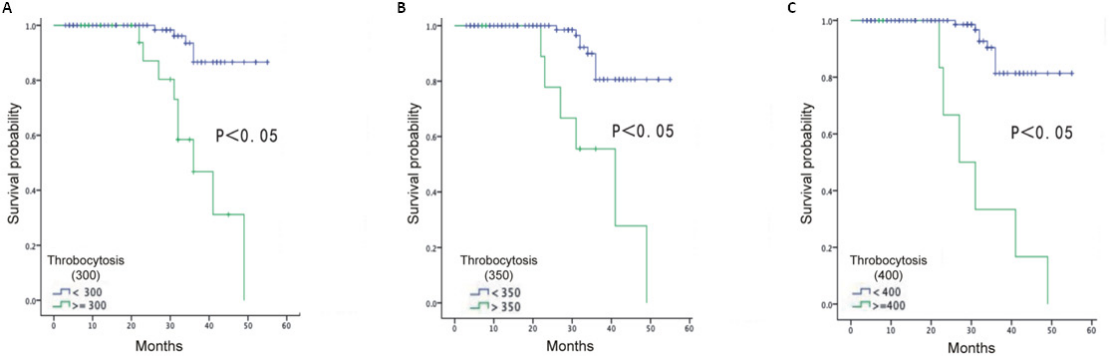
Overall survival of EOC patients based on definition of thrombocytosis at 300×10^9^/L **(A)**, 350×10^9^/L **(B)** and 400×10^9^/L **(C)**.

We also studied the preoperative platelet count at different levels to determine the most optimal cutoff value to predict an optimal surgery at 232.2 × 10^9^/L, advanced disease stage at 208.5 × 10^9^/L and overall survival at 327.5 × 10^9^/L (Supplementary Table 3). From the table, a preoperative platelet count of 232.2 × 10^9^/L yielded the most optimal predictive value to predict a suboptimal surgery. The AUC for determining a suboptimal surgery was 0.62 (95% confidence interval (CI), 0.53 to 0.71), whereas the sensitivity, specificity, positive predictive value (PPV), and negative predictive value (NPV) were 62.5%, 57.39%, 42.61%, and 37.5%, respectively. A preoperative platelet count of 208.5 × 10^9^/L yielded the best predictive values for advanced-stage disease. The AUC was 0.62 (95% CI, 0.54 to 0.71), whereas the sensitivity, specificity, PPV and NPV were 73.17%, 45.24%, 54.7% and 26.83%, respectively. To determine overall survival, a preoperative platelet count of 327.5 × 10^9^/L exhibited an AUC of 0.80 (95% CI, 0.66 to 0.95), a sensitivity of 64.29%, specificity of 93.75%, PPV of 6.25% and NPV of 35.71%. All the EOC patients were followed for a median of 30 months (3-55 months). When using the different preoperative platelet cutoff values of 300 × 10^9^/L, 350 × 10^9^/L and 400 × 10^9^/L, the EOC patients with thrombocytosis exhibited a significantly shorter overall survival time (median: 37.5. months; 36.4 months; 32.1 months, respectively) compared with those with platelet counts less than 300 × 10^9^/L, 350 × 10^9^/L and 400 × 10^9^/L (median: 52.1 months; 50.8 months; 51.0 months, respectively; p < 0.0001) (Figure 2A–2C). Thus, high platelet levels could predict an advanced stage, optimal surgery and overall survival.

### PMPs induce proliferation and migration of ovarian cancer cells

**Figure 3:**
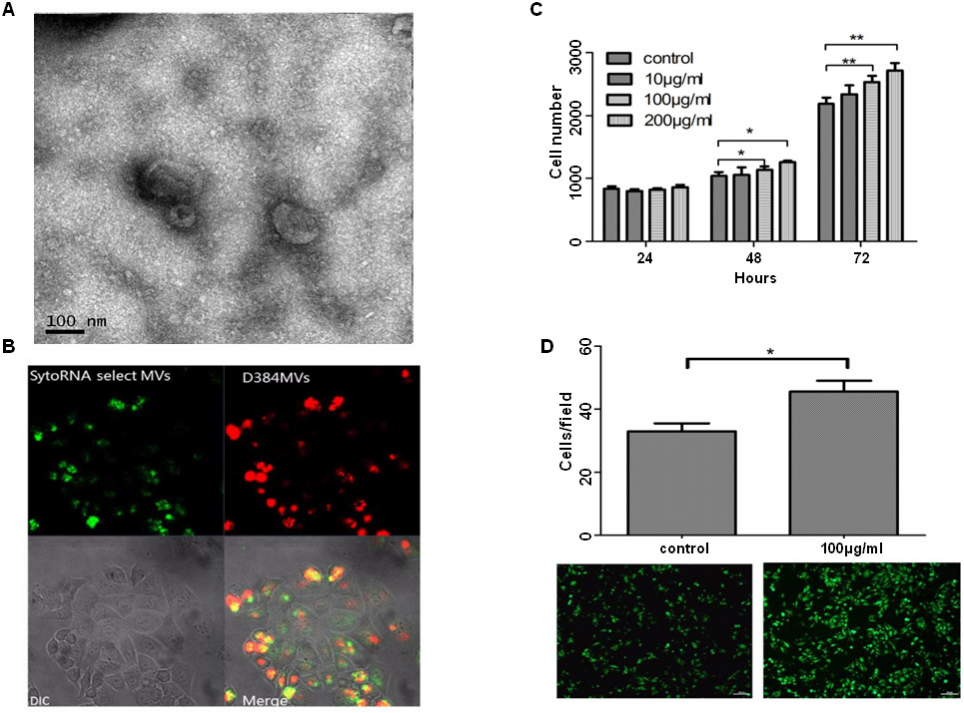
PMPs induce proliferation and migration of ovarian cancer cells **(A)** Scanning electron microscope (SEM) of PMPs. The size of PMPs is probably ranging from 0.05μm to 0.8μm. **(B)** Confocal images of PMPs uptake by SKOV3 cells. PMPs stained with D384, a phospholipids membrane dye (red stain; top left panel) and SytoRNA Select, an RNA-select stain (green stain; bottom left panel). PMPs (100μg/ml) are incubated with unstained naive recipient cells for 12 hours. **(C)** The proliferation rate of SKOV3 cells increase significantly after co-incubation with PMPs depending on the concentration. **(D)** The effects of purified PMPs (100μg/ml) on migration of SKOV3 cells are determined by using 24 well Transwell Permeable Supports with 8 μM pores. Values of relative fluorescence are shown. n=3. ^*^p < 0.05 compared to control. ^**^p < 0.01 compared to control. Scale bar, 100μm.

The collected platelets from the blood of healthy human donors that were stimulated with thrombin to obtain PMPs were analyzed. The quantity of protein was quantified using the BCA method [12]. Using a scanning electron microscope (SEM) to visualize isolated PMPs and determine their structure, the size of PMPs probably ranges from 0.05μm to 0.8μm (Figure 3A). To determine the mechanism of PMPs uptake by SKOV3 cells, we co-cultured the PMPs and SKOV3 cells and observed the uptake process using confocal microscopy. The fluorescent PMPs and transferred RNA were apparent in the recipient cell membranes and cytoplasm by the appearance of the red dye and green stain, respectively (Figure 3B). SKOV3 cells treated with concentrations of PMPs ranging from 10 to 200μg/ml were cultured for 3 days to study their proliferation. The results revealed that the growth of the cells increased significantly over time and with higher PMPs concentrations (Figure 3C). We also observed that the PMPs promoted the migration of the SKOV3 cells by approximately 15% (Figure 3D). Consequently, the PMPs were internalized by endocytosis and promoted the proliferation and migration of the cancer cells.

### PMPs enhance the metastasis of EOC by inducing EMT

**Figure 4:**
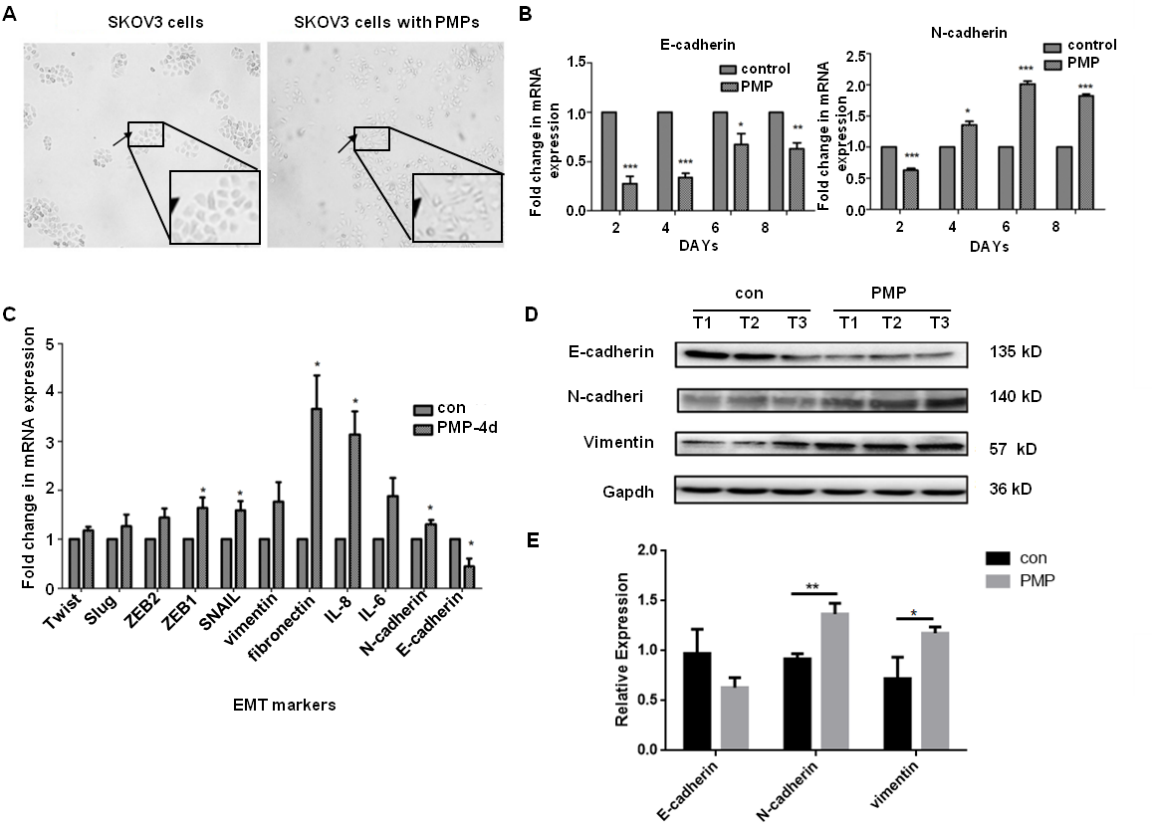
PMPs induce EMT of Ovarian cancer cells **(A)** SKOV3 cells are stimulated for 48 hours with PMPs (100μg/ml). Photomicrographs of SKOV3 cells show a rounded epithelial cell shape (left panel) and a fibroblastic-type phenotype when cocultured with PMPs (right panel); original magnification, ×40. **(B)** Real-time PCR analysis of E-cadherin and N-cadherin at different time. **(C)** Real-time PCR analysis of transcription factors and EMT-specific genes in SKOV3 cells co-cultured with PMPs for 4 days. The relative mRNA levels were normalized to β-actin. **(D, E)** Western blot analysis of EMT-specific proteins in SKOV3 cells co-cultured with PMPs for 8 days. Gapdh served as the loading control. T1, T2 and T3 stand for the first, second and third time. n=3. ^*^P < 0.05. ^**^P < 0.01, ^***^P<0.001.

PMPs can promote tumor metastasis. However, the mechanism remains unclear. The cancer cells co-cultured with 100 μg/ml PMPs were analyzed for mesenchymal markers and transcription factors involved in EMT. E-cadherin was down-regulated, whereas N-cadherin, vimentin, snail, fibronectin, MMP-2, MMP-9, and ZEB2 were up-regulated. The results indicate that the ovarian cancer cells with PMPs tended to spindle and exhibit a fibroblastic-type phenotype (Figure 4B). The expression of E-cadherin obviously decreased at an early stage, and increased expression of N-cadherin followed (Figure 4B). Next, we co-cultured the cells for 4 days and analyzed other factors associated with EMT. Twist, Slug, ZEB2, ZEB1, snail, vimentin, fibronectin, interleukin (IL)-8, and IL-6 also increased. The RNA and protein levels indicated that EMT had occurred in the cancer cells (Figure 4C and 4D). Thus, PMPs could induce EMT in ovarian cancer cells.

### miR-939 promotes the epithelial-mesenchymal transition in ovarian cancer

**Figure 5:**
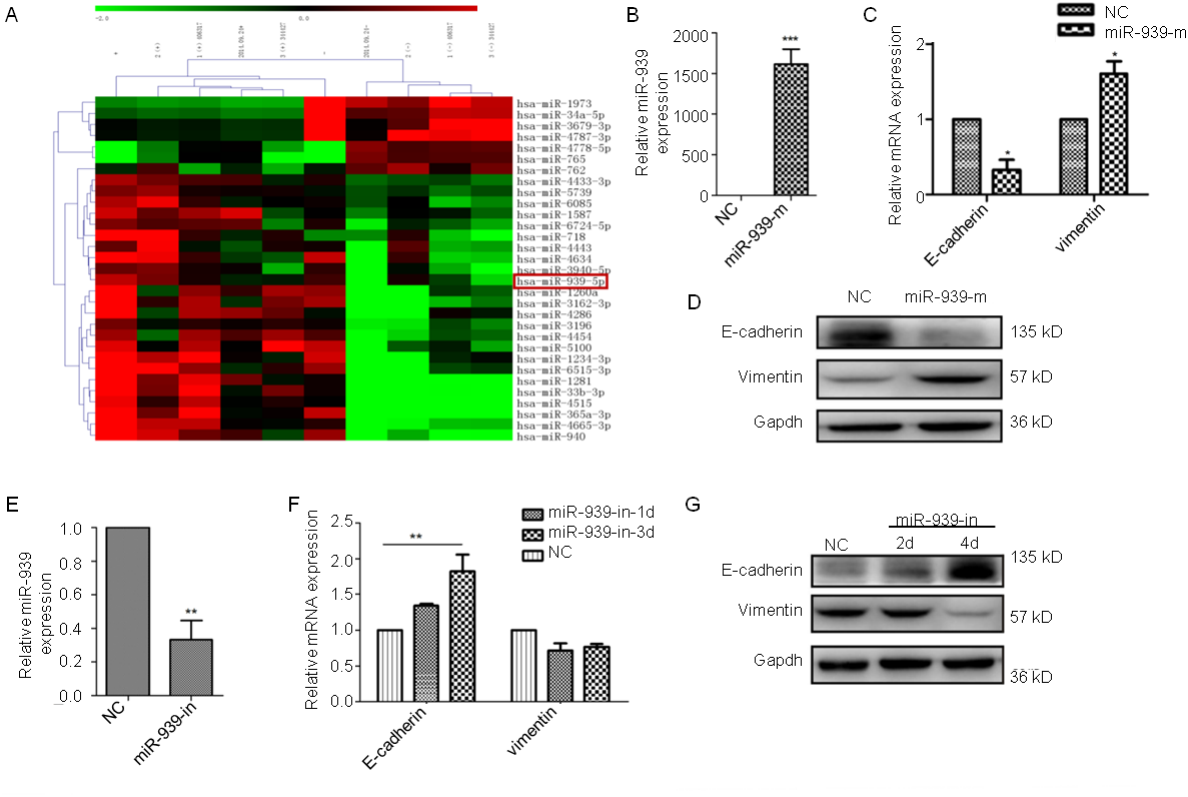
miR-939 promotes EMT in ovarian cancer **(A)** Heatmap of the altered miRNA expression in PMPs from platelets stimulated by thrombin or apoptosis. Validation of miR-939 expression levels after transfecting miR-939 mimics(miR-939-m) **(B)** or inhibitor (miR-939-in) **(E)** by PCR analysis. Real-time PCR analysis of the EMT-specific genes in SKOV3 cells transfected miR-939 mimics **(C)** or miR-939 inhibitor after 1(miR-939-in-1d) or 3(miR-939-in-3d) days **(F)**. The relative mRNA levels were normalized to β-actin. Western blot analysis of E-cadherin and vimentin protein in SKOV3 cells transfected miR-939 mimics **(D)** or inhibitor **(G)**. Gapdh served as the loading control. NC means normal control. n=3. ^*^P < 0.05.

Next, we used a significance microarray analysis and analyzed the miRNA differences between the two types of PMPs from the platelets stimulated by thrombin or apoptosis (Figure 5A). After consulting the literature, we found that in addition to miR-939, other miRNAs with obvious differences have no significant correlation with tumor. Therefore, we hypothesized that miR-939 in PMPs would release and activate cancer cells after being absorbed by ovarian cancer cells, thus promoting the progression of ovarian cancer. In the SKOV3 cells transfected with miR-939 mimics, miR-939 inhibitor or the respective controls, the expression of miR-939 was verified by qRT-PCR (Figure 5B and 5E). The expression of mRNAs translated after 24 hours is related to EMT-specific genes changes. The protein and mRNA levels of E-cadherin decreased, whereas vimentin, fibronectin, ZEB2 and slug expression increased (Figure 5C and 5D). The miR-939 knockdown in the cancer cells resulted in epithelial cell features. The expression of E-cadherin was high-expressed, whereas vimentin expression decreased as time progressed. The related proteins exhibited more obvious changes (Figure 5E and 5F). In addition, we also found miR-939 could improve the proliferation and migration of ovarian cancer cells (Supplementary Figure 2). Therefore, miR-939 might be the key element that mediates the interaction between platelets and cancer cells, thereby promoting metastasis though EMT.

### sPLA_2_-IIA induces a combination of PMPs and ovarian cancer cells

**Figure 6:**
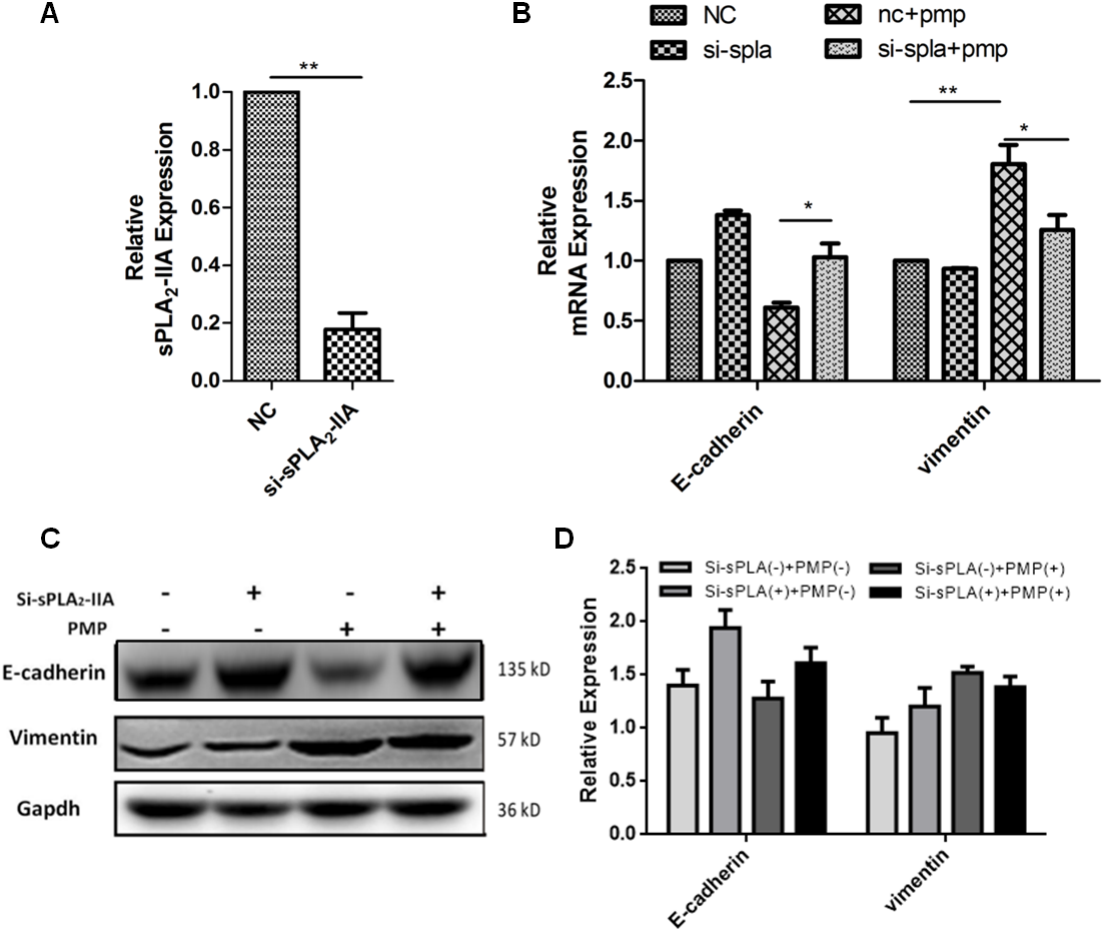
sPLA2-IIA induces the internalization of PMPs by ovarian cancer cells **(A)** Validation of sPLA2-IIA expression levels after transfection by PCR analysis **(B-C)** Knockdown sPLA2-IIA in SKOV3 by siRNA, then co-cultured with PMPs for 4 days. Real-time PCR analysis of the change of EMT-specific genes in SKOV3 cells (B). The relative mRNA levels were normalized to β-actin. Western blot analysis of E-cadherin and vimentin protein in SKOV3 cells (C, **D**). Gapdh serves as the loading control. n=3.

PMPs can be internalized by neutrophils through sPLA_2_-IIA [10]. We hypothesized that sPLA_2_-IIa induces the combination of PMPs and ovarian cancer cells. sPLA_2_-IIA (Figure 6A) was knocked down in the SKOV3 cells via siRNA. The resulting SKOV3 cells were co-cultured with PMPs for 4 or 8 days, and mRNA and protein levels were assessed at both time points. The results revealed that E-cadherin was increased when PLA_2_-IIA was knocked down in the SKOV3 cells. In the co-culture of PMPs and sPLA_2_-IIA knockdown SKOV3 cells, E-cadherin expression increased. However, vimentin expression decreased (Figure 6B and 6C). Thus, sPLA_2_-IIA could promote the process of internalization of PMPs by ovarian cancer cells.

## DISCUSSION

Although platelet and ovarian cancer have been studied for many years, our recent study analyzed novel features of their interaction. First, using a large amount of clinical data, we revealed the clinical significance of preoperative platelet aggregates in the progression of EOC. Second, we were the first to illuminate that PMPs induce the EMT of ovarian cancer by acting as the carrier in platelet-tumor cell crosstalk. Third, to date, no studies have demonstrated that miR-939 is associated with EMT. Fourth, we found that sPLA_2_-IIA could promote PMPs uptake by cancer cells, which is not often studied.

The prevalence of thrombocytosis in patients with ovarian cancer ranges from 22.3% to 68% [4, 13–15]. Generally, the cutoff value of thrombocytosis associated with cancer was as a platelet count at least 400×109/L or 450×10^9^/L in other studies. Only 7.0% of patients exhibited platelet counts greater than 400^*^109/L in this study. Another published study in China by Qiu, J et al. [16] found that 7.4% (10/136) of EOC patients had thrombocytosis with platelet count >400 × 10^9^/L, which was similar to our results. However, one study from Korean suggested that the incidence of thrombocytosis in EOC was 36.4% if more than 400×10^9^/L was selected as the cutoff [17]. The authors suggested that the incidence of thrombocytosis varies among population from different Asian countries. In addition, in our study using the ROC curve, preoperative platelet count of 327.5*10^9^/L, which was less than 350×10^9^/L, yielded the most optimal predictive value to predict overall survival. Therefore, thrombocytosis was defined as a platelet count great than 350×10^9^/L, and the incidence of thrombocytosis with EOC was 11.7% in this study.

Thrombocytosis has been consistently shown to be associated with advanced-stage and higher grade cancers, more frequent lymph node metastases, greater volumes of ascites, and reduced overall survival [15, 18, 19]. Some of our results were consistent with previous studies. In this study, the presence of thrombocytosis suggested that the platelet count in EOC was positively related to the CA125 level (p = 0.049), larger postoperative residue tumor volumes, more ascites formation, more organ involvement, surgical staging and poorer survival. Thus, anti-thrombosis treatments, including heparin [20–24] and anti-interleukin 6 antibody [4], in cancer patients could prolong survival by interfering with the crosstalk between tumors and platelets.

PMPs were regarded as inert “platelet dusts” with a potential role in the platelet-cancer loop. PMPs are the most abundant microparticles in the blood, constituting the majority (> 80%) of the pool of MPs [25]. PMPs transport miRNAs, growth factors and other bioactive molecules and participate in forming platelets [12, 18]. PMPs are also involved in the cascade of cellular injury and vascular dysfunctions underlying thrombosis, especially in cancer [26]. PMPs promote invasion of prostate cancer cells by up-regulating MMP-2 [27]. PMPs also promote proliferation, differentiation, and survival of neuronal cells, indicating a role in treating brain injuries. Probable mechanisms for this activity likely involve the pAKT and protein kinase RNA-like endoplasmic reticulum kinase (pERK) pathways [28]. As previously mentioned, we also found that PMPs promote ovarian cancer cell proliferation and migration and the expression of EMT-specific genes and promote EMT in ovarian cancer. However, the dynamic changes and special markers of tumor-associated PMPs in the tumor microenvironment, blood, and other regions, such as urine, have not been thoroughly explored. Understanding their mechanism has great value in early clinical screening and diagnosis.

Our microRNA microassay of PMPs derived from platelets stimulated by thrombin or naturally by apoptosis revealed a significant difference in miR-939. miR-939 is up-regulated in cancers, such as lung adenocarcinoma [29, 30] and high-risk neuroblastoma [31]. miR-939 regulates a large number of genes, such as WNT1, NTSR1, POLK, WWOX and SPN, that are related to lung cancer [32]. In ovarian cancer, miR-939 dramatically promoted ES-2 cell proliferation by suppressing APC2 expression [30, 33]. Given the results from our study, miR-939 may be the key factor in PMPs that induces the EMT. As expected, miR-939 levels were high in the PMPs. miR-939 down-regulated E-cadherin and up-regulated vimentin. Also miR-939 could stimulate the proliferation and migration of ovarian cancer cells. These results indicate that miR-939 could induce EMT and promote tumor progression, which is a novel finding. About its mechanism, we found that miR-939 promoted EMT of ovarian cancer cell with E-cadherin down-regulated. And there is a predicted consequential pairing of target region between miR-939 and the 3`UTR of E-cadherin (position 802-808). Moreover, miR-939 has several potential target regions with the 3`UTR regions of claudin (e.g. position 71-77). Based on these target region analysis, miR-939 might promote EMT via down-regulating EMT markers E-cadherin and claudin [34, 35]. Given the importance of EMT in the development of tumors and that the role of cell signaling pathways are unclear, further research is necessary to determine the mechanism of miR-939 and provide new targets for cancer treatment.

Although PMPs are accepted as an important means of intercellular communication, the mechanisms underlying PMPs internalization in recipient cells was poorly understood until Eric Boilard et al. found that 12(S)-hydroxyeicosatetranoic acid (12(S)-HETE) and sPLA_2_-IIA activate the internalization by neutrophils [10]. sPLA_2_-IIA is one of the oldest identified and studied enzymes. It is involved in various life-threatening diseases, such as asthma, diabetes, autoimmune disorders, rheumatoid arthritis, several neurological disorders [36], stable coronary heart disease [37] and various types of cancers (lung adenocarcinoma [38], gastric cancer [39], primary resected esophageal squamous cell carcinoma, and prostate cancer [40]. sPLA_2_-IIA inhibition is considered an important therapeutic approach for different inflammatory diseases [41]. sPLA_2_-IIA is expressed by platelets in inflammation, as well as by cancer cells [40]. In our study, we found that sPLA_2_-IIA mediated the internalization of PMPs by ovarian cancer cells, which has not yet been reported. Given the effects of sPLA_2_-IIA in cancer cell internalization of PMPs, we believe that sPLA_2_-IIA could assist in tumor diagnosis and treatment in the near future.

Our study indicated that high levels of platelets could enhance the progression of EOC and lead to poor prognosis (Supplementary Figure 1). miR-939 might be an oncomiR and a new target for ovarian cancer treatment. Moreover, inhibiting the function of sPLA_2_-IIA may offer a new treatment for ovarian cancer. Ovarian cancer is difficult to diagnosis in the early stages. Therefore, new diagnostic markers are necessary. Perhaps in the near future, ovarian cancer diagnoses can be made using PMPs in the blood or urine. Therapiestargeting PMPs, miR-939 or sPLA_2_-IIA could significantly inhibit the progression of tumors and improve patient prognosis.

## MATERIALS AND METHODS

### Clinical data

From Nov 2008 to Jun 2013, 448 patients with an ovarian tumor who were undergoing treatment at Shanghai First Maternity and Infant Hospital were enrolled in this study. This group included 218 patients with benign epithelial ovarian tumors, 59 patients with borderline ovarian tumors with feature of lower malignant potential and good prognosis and 171 patients with EOC. This study was approved by the Institutional Review Board of the Shanghai First Maternity and Infant Hospital. Patients were excluded if they had incomplete preoperative laboratory data or if they underwent neoadjuvant therapy, suffered recurrent EOC or did not consent to the use of their medical records for research. For all EOC patients underwent debulking surgery and followed by chemotherapy based platinum. The median followup was at median 30 months (3-55 months). 123 patients obtained optimal surgery with less than 1 cm residue size tumor, the other 48 patients were suboptimal surgery.

### Clinical and laboratory data collection

The predictive values studied included the area under receiver operating characteristic (ROC), sensitivity, specificity, positive predictive value (PPV), and negative predictive value (NPV). Optimal surgery was defined as surgery resulting in residual disease after debulking less than 1 cm. Overall survival (OS) was assessed from diagnosis to the date of death from any cause or to the date of last contact.

### Microparticles

#### Platelet microparticles

Platelets were derived from citrated blood of healthy human donors under an Institutional Review Board-approved protocol. Platelets were isolated after centrifugation of the blood (1200 r for 30 min at 21°C). Supernatant (platelet-rich plasma) was centrifuged at 2000 r for 30 min at 21°C. Pellet-containing platelets were resuspended in RPMI-1640 medium (HyClone, Logan, UT). Platelets were counted (Clinical Laboratory, Shanghai First Maternity and Infant Hospital, Shanghai) and adjusted to a density of 150 × 10^6^ cells/ml before they were supplemented with 1.5% Citrate-dextrose solution (ACD, Sigma, Sigma-Aldrich, St Louis, MO) and stimulated with thrombin (1.0u/ml; Takeda Austria) for 1 hour. After centrifugation at 4000 r for 10 min at 4°C, the supernatant containing the PMPs was then centrifuged at 50,000 × g for 60 min at 4°C. Pellets containing PMPs were resuspended in RPMI-1640 medium and quantified using the BCA method.

### Cell lines

Human ovarian cancer cell line SKOV3 was purchased from the American Type Culture Collection at December 2013. The cell line was authenticated by STR test, and the cell line was tested in December 2015.

### Treatment of tumor cells with PMPs

Cells were seeded in RPMI-1640 medium (HyClone, Logan, UT) with 10% FBS (FBS, Gibco, USA) and 1% penicillin-streptomycin (P/S, Gibco, USA) for 8 hours then started using empty medium for 4 hours. The medium was changed with 2% FBS 1640. PMPs were added at a final concentration of approximately 100μg/ml.

### Cell proliferation analysis

Approximately 3000 cells/well of SKOV3 cells were seeded in 96-well plates with difference concentrations of PMPs. Cultures were stained after 1, 2, and 3 days. Culture absorbance was measured at 490 nm in a Thermo Scientific Multiskan (Thermo Fisher Scientific, USA) after incubating the cells with 20μL of a 5 mg/ml 3-(4,5-dimethyl-2-thiazolyl)-2,5-diphenyl-2-H-tetrazolium bromide (MTT, Sigma–Aldrich) solution at 37°C for 4 hours. The resulting MTT formosan was solubilized in dimethyl sulfoxide (DMSO).

### Migration assays

For transwell migration assays, 1×10^4^ cells were plated in the top chamber of a transwell containing a non-coated membrane (24-well insert; 8-mm pore size; BD Biosciences). Cells were plated in serum-free medium. Medium supplemented with 10% (v/v) serum was used as a chemoattractant in the lower chamber. The experimental group was treated with 100μg/ml PMPs and incubated for 16 hours at 37°C in a culture incubator with 5% (v/v) CO2. Cells that migrated to the lower sides of the inserts were stained with Giemsa and counted.

### miRNA microarray

Total miRNA expression of PMPs from platelets stimulated by thrombin or apoptosis were analyzed by Agilent’s miRNA Complete Labeling and were identified on the Gene Expression Omnibus (http://ncbi.nlm.nih.gov/geo, Design ID: 046064) website. Culture platelets in the empty medium for 48h for natural apoptosis.

### Gene knockdown using small interfering RNA

Small interfering RNAs for miR-939 (inhibitor) and sPLA_2_-IIA (siRNA) were synthesized by Bioneer technology (Shanghai Genepharma Co., Ltd. Shanghai, CA). Cells were transfected with siRNA or inhibitor at a final concentration of 100 nM using lipofectamine (Invitrogen), according to the manufacturer’s suggested protocol. Gene knockdown was then verified by real-time quantitative polymerase chain reaction (qRT-PCR).

### Transient cell transfections

miR-939 mimics, a negative control and miR-939 inhibitor were purchased from GenePharma Co, Ltd (Shanghai, CA). These agents were transfected into SKOV3 cells using Lipofectamine_2000 reagent (Invitrogen) according to the manufacturer’s instructions. Validation of miR-939 expression levels after transfection was performed using a PCR analysis.

### RNA extraction and real-time quantitative PCR

Total RNA including microRNAs was extracted from culture cells using Trizol reagents and treated with DNaseI. qRT-PCR were performed using the Two-Step PrimeScript miRNA cDNA Synthesis Kit (Takara, Dalian, China) using a ABI 7500 Real Time PCR system (Applied Biosystems, Foster City, CA, USA). Relative miRNA expression levels after normalization to U6 small nuclear RNA were calculated using 2 [(Ct of miR-939) (Ct of U6)]. qRT-PCR was performed by SYBR Kit (Qiagen, China) using a Light Cycler system. Relative mRNA expression levels were normalized to the geometric mean of β-actin to control for variability in expression levels and calculated.

### Western blotting

The procedure used was referenced from Gillanders et al. [42] using a Bicinchoninic Acid (BCA) Protein Assay kit (Beyotime, China) to measure protein concentration. Primary antibodies (anti-E-cadherin, anti-N-cadherin, anti-vimentin, anti-slug and anti-snail) were purchased from Cell Signaling Technology, and Gapdh antibody (Sigma-Aldrich) was used as a normalization control. The secondary antibody was a horseradish peroxidase-conjugated antibody (Sigma–Aldrich) that was incubated for 1 hour at room temperature. Enhanced chemiluminescence (ECL) was used to visualize immunocomplexes following the manufacturer’s protocol.

### Confocal microscopy

PMPs were stained with D384, a phospholipid membrane dye (red stain), and SytoRNA Select, an RNA-select stain (green stain). Then, 100 μg/ml PMPs was incubated with unstained naive recipient cells for 12 hours.

### Statistical analysis

SPSS ver. 20.0 (SPSS Inc., Chicago, IL, USA) and GraphPad Prism Software (version) were used for statistical analyses. Data from the different groups were compared using a chi-square test or Fisher’s exact test. OS was calculated using a Kaplan-Meier method to determine the univariate relationship of thrombocytosis. ROC curves were plotted and the areas under the curve(AUC) were calculated for comparison. The ROC curves were generated by plotting the relationship of the true positivity (sensitivity) and the false positivity (1-specificity) at various cut off points of the tests. A p-value < 0.05 was considered statistically significant.

## SUPPLEMENTARY MATERIALS FIGURES AND TABLES


