# Supplementary material for: Platelet microparticle-mediated transfer of miR-939 to epithelial ovarian cancer cells promotes epithelial to mesenchymal transition
Source: Oncotarget. 2017 Oct 27;8(57):97464–75. doi: 10.18632/oncotarget.22136 (PMC5722576; doi:10.18632/oncotarget.22136)
Supplement: Supplementary file 1 [file oncotarget-08-97464-s001.pdf]

Platelet microparticle-mediated transfer of miR-939 to epithelial ovarian cancer cells promotes epithelial to mesenchymal transition

SUPPLEMENTARY MATERIALS

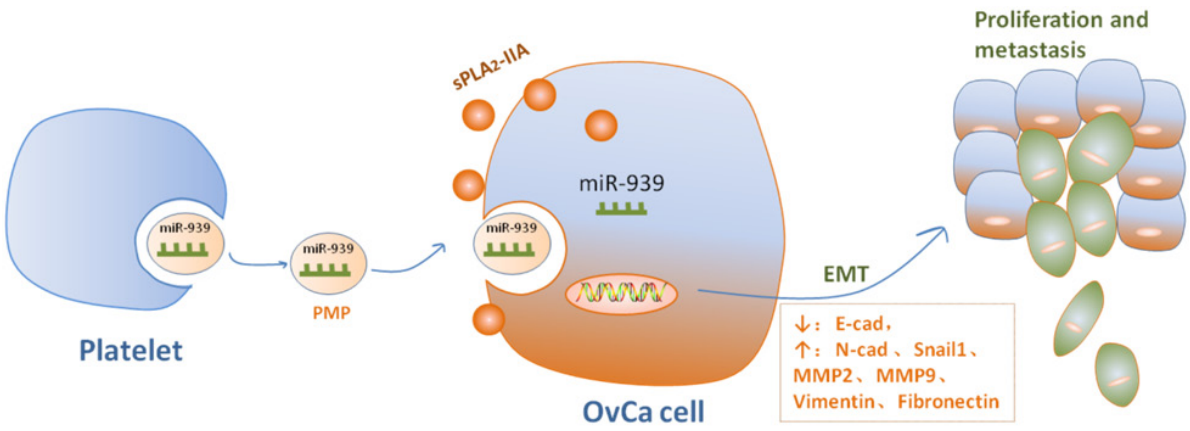

Supplementary Figure 1: A schematic diagram of platelets microparticles(PMPs) regulation by miR-939 in promoting tumor metastases. In tumor microenvironment, ovarian cancer cells secrete sPLA2-IIA to induce binding with the PMPs. PMPs once uptaken, the miRNAs, especially miR-939, make epithelial-mesenchymal transition (EMT) and promote the proliferation and metastasis in ovarian cancer.

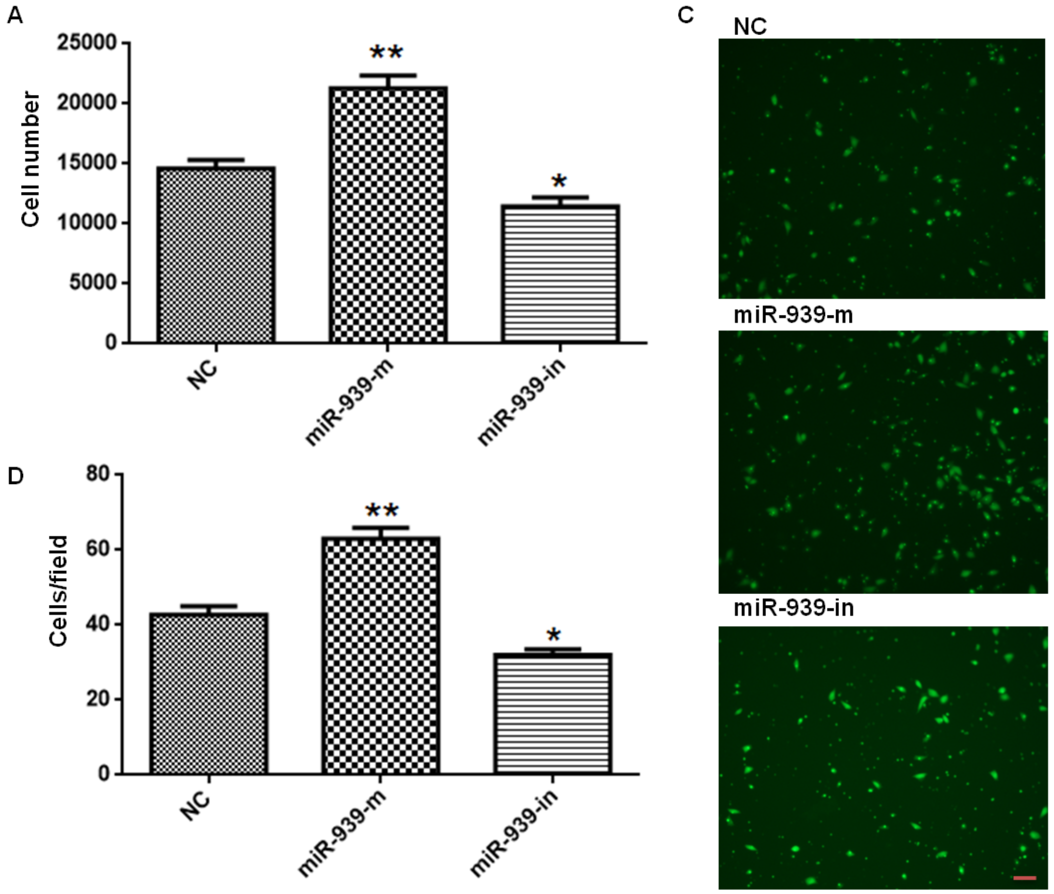

Supplementary Figure 2: miR-939 could improve the proliferation and migration of ovarian cancer cells. The proliferation rate **(A)** and the migration **(B, C)** of SKOV3 cells transfecting miR-939 mimics(miR-939-m) or inhibitor (miR-939-in) are shown. n=3. * p < 0.05 compared to control. ** p< 0.01 compared to control. Scale bar, 100μm.

Supplementary Table 1: Characteristics of patients with benign, borderline and malignant epithelial of ovarian tumor (Mean±SD)

Characteristics		Benign (n=218)	Borderline (n=59)	Malignant (n=171)	*P-value*
Age		39.8±14.4	41.2±13.7	51.4±11.5	<0.001
PLT		188.3±49.1	206.9±80.5	248.0±96.8	<0.001
PT (s)		16.8±1.2	15.4±3.1	14.1±3.1	<0.001
Platelet ≥350*10^9/L		1(0.5%)	2(3.4%)	20(11.7%)	<0.001
<350*10^9/L		217(99.5%)	57(96.6%)	151(88.3)	
≥400*10^9/L		1(0.5%)	0(0.0%)	12(7.0%)	<0.001
<400*10^9/L		217(99.5%)	59(100.0%)	159(93.0%)	
Residual tumor size	<1.0cm	218(100%)	58(98.3%)	114(66.7%)	<0.001
	≥1.0cm	0(0.0%)	1(1.7%)	57(33.3%)	
Preoperative platelet / postoperativeplatelet (Mean±SD)		1.1±0.2	1.1±0.38	1.3±0.3	<0.001
Preoperative CA125/ postoperative CA125 (Mean±SD)		/	4.2±9.6	2.27±2.7	0.144

Supplementary Table 2: Comparison of EOC patients with thrombocytoisis and non-thrombocytosis (Mean±SD)

Characteristics		PLT<350 (n=151)	PLT≥350 (n=20)	*P-value*
Age		51.4±11.8	51.5±8.5	0.976
Preoperative	PT	14.1±3.0	14.2±3.9	0.921
Preoperativetumor markers	CA125	362.1±400.3	473.5±378.7	0.242
	CA199	141.9±305.8	70.5±151.7	0.320
	AFP	21.4±113.7	6.1±8.3	0.570
	CEA	7.3±30.3	6.6±22.6	0.916
Preoperative platelet/ postoperative platelet	<2	143(98.6%)	17(89.5%)	0.066
Preoperative CA125/ postoperative CA125	<2	69(56.1%)	5(38.5%)	0.254
CA125 after 3th CTX	<35	107(89.9%)	11(84.6%)	0.629
Preoperative platelet / platelet after 3th CTX	<2	98(84.5%)	9(60.0%)	0.032
	≥2	18(15.5%)	6(40.0%)	
Clinical symptom				
Syndrome	Abdominal floating	51(33.8%)	10(50.0%)	0.213
	Abdominal pain	40(26.5%)	13(65.0%)	0.001
	Ascites	15(9.9%)	4(20.0%)	0.245
	Low back pain	9(6.0%)	3(15.0%)	0.151
	Volume of ascites	35.9±27.1	57.3±33.2	0.029
	Anemia	6(4.0%)	3(15%)	0.073
Site of mass	Unilateral	108(71.5%)	11(55.0%)	0.107
Site of mass	Bilateral	43(28.5%)	9(45.0%)	
Surgical pathology finding				
Residual tumor size	<1cm	107(70.9%)	8(40.0%)	0.010
Residual tumor size Stage	≥1cm	44(29.1%)	12(60.0%)	
	I-II	79(53.7%)	5(26.3%)	0.029
	III-IV	68(46.3%)	14(73.7%)	
Grade	G1	28(26.2%)	1(7.7%)	0.314
	G2	24(22.4%)	4(30.8%)	
	G3	55(51.4%)	8(61.5%)	
Involvement of other organs	Cervical canal	9(6.0%)	0(0.0%)	0.825
Involvement of other organs Num of positive lymph nodes	Omentum	48(31.8%)	13(65.0%)	0.006
	Intestinal tract	44(29.1)	10(50.0%)	0.074
	liver	11(7.3%)	4(20.0%)	0.080
	Diaphragmatic surface	10(6.0%)	5(25.0%)	0.018
	peritoneum	67(44.4%)	15(75.0%)	0.016
		0.7±2.3	1.2±2.68	0.653

Supplementary Table 3: The association between the stage, optimal surgery and the preoperative plates level

		Preoperative platelets (ROC cutoff value)			p- value	AUC	95%CI	Sens (%)	Spec (%)	PPV (%)	NPV (%)
		≥208.5	<208.5	SUM							
Stage	III+IV	60	22	82							
	I+II	46	38	84	0.014*	0.62	0.54-0.71	73.17	45.24	54.76	26.83
	SUM	106	60	166							
		≥232.2	<232.2	SUM							
Surgery	Subotimal	35	21	56							
	Optimal	49	66	115	0.015*	0.62	0.53-0.71	62.5	57.39	42.61	37.5
	SUM	84	87	171							
		≥327.5	<327.5	SUM							
Survival	die	9	5	14							
	live	7	105	112	0.000*	0.80	0.66-0.95	64.29	93.75	6.25	35.71
	SUM	16	110	126							

AUC= Area Under roc Curve, CI= confidence interval, Sens= Sensitivity, Spec= Specificity, PPV=positive predictive value, NPV=negative predictive value.
